# Frequency of Anemia and Iron Deficiency among Children Starting First Year of School Life and Their Association with Weight and Height

**DOI:** 10.1155/2018/8906258

**Published:** 2018-04-10

**Authors:** Mirza Sultan Ahmad, Hadia Farooq, Sumaira Noor Maham, Zonaira Qayyum, Abdul Waheed, Waqar Nasir

**Affiliations:** Fazle Omar Hospital Rabwah, Chenab Nagar, Pakistan

## Abstract

The objectives of the study were to ascertain frequency of anemia and iron deficiency among children starting first year of school life and test association with height and weight. One in four children starting first year of school life in five schools of Rabwah, Pakistan, was included. Full blood counts and ferritin levels of the children included in the study were checked. Status of their height and weight was determined according to *Z*-score charts. Chi-square test was used to test association. Two hundred and ninety-five children with median age of 67 months were included in the study. Out of 295, 240 (81.4%) had normal Hb and 55 (18.6%) had anemia. Ferritin levels were found to be below normal level in 242 (82%) children. There was no significant difference between hemoglobin and ferritin levels of children belonging to different categories of height and weight. Spearman test showed that there was very weak correlation between ferritin and hemoglobin levels (*r*_*s*_ = .163). Our conclusions were that iron deficiency without anemia is very frequent among children starting first year of school. Regression models show that ferritin levels cannot be predicted by independent variables like status of height and weight on *Z*-score charts, age, gender, and anemia.

## 1. Introduction

Iron deficiency is the commonest nutrient deficiency in the world and a major public health risk in both the developing and industrialized countries. It affects more than a billion people of different age groups around the world [1–4]. It is the commonest cause of anemia and is also a common deficiency among nonanemic children, especially among children of resource limited countries. A study by Ekwochi et al. showed that iron deficiency was present in 27.5% of nonanemic children under 5 [5]. Iron is necessary for healthy function and development of brain. There is evidence that its deficiency without anemia causes fatigue. It can affect visual and auditory functioning and is weakly associated with poor cognitive development in children [6–9].

This makes it important to study frequency of iron deficiency among children starting the first year of school. Serum ferritin is the preferred initial diagnostic test for iron deficiency. It depicts the status of iron stores in body [10, 11]. The first year of school life is important because it is the start of academic career. Usually children start their school life at 5 years age. Like any other age group different nutritional problems are common at this age.

The objectives of this study were

1. To ascertain the frequency of iron deficiency and anemia among the children starting first year of school
2. To ascertain whether value of dependent variable, that is, ferritin, can be predicted based on independent variables like age, gender, anemia, status of height according to WHO (World Health Organization) *Z*-score charts, and status of weight according to WHO *Z*- score charts.

## 2. Methods

Five schools, belonging to the largest school system of the town, that is, Nazarat Taleem school system, out of a total of 11 schools providing primary level education in Rabwah were selected for this study. One in four children was selected by computer randomization. The children having fever and any sign or symptom of infectious disease or inflammation and the children receiving iron therapy were excluded from the study. Names of all children admitted in prep class (first year of school) were entered in software, and one in four children was selected by computer randomization. Five milliliter of the blood of the selected children was drawn for full blood count and ferritin levels. Full blood count (FBC) was checked by Medonic M 20 analyzer, and ferritin levels were checked by Elisa method (Statfox 200). Name, age in months, sex, hemoglobin levels (Hb), and ferritin levels were entered in a data form and pro forma. SPSS 20 was used for data analysis. For children below 5 years of age hemoglobin (Hb) level of <11.0 was categorized as anemia, and for children > 5 years of age Hb level <11.5 gm/dl was labeled as anemia. For children below 5 years of age ferritin levels below 12 ng/ml were labeled as low ferritin levels and for children above 5 years of age ferritin level <10 ng/ml was defined as low ferritin level. Those with both hemoglobin and ferritin levels below normal were categorized as having iron deficiency anemia.

Weight and height of these children were checked with a single combined height and weight scale of RE 160 type. These parameters were plotted on World Health Organization (WHO) *Z*-score charts for height and weight. WHO charts were used for male and female children of different ages included in this study: (i) *Z*-score weight for age chart for boys from birth to 5 years of age; (ii) *Z*-score weight for age chart for boys from 5 to 10 years; (iii) *Z*-score weight for age chart for girls from birth to 5 years; (iv) *Z*-score weight for age charts for girls from 5 to 10 years; (v) *Z*-score height for age chart for boys from birth to 5 years; (vi) *Z*-score height for age charts for boys from 5 to 19 years; (vii) *Z*-score height for age chart for girls from birth to 5 years; (viii) *Z*-score height for age chart for girls from 5 to 19 years. These charts were used to categorize weight for age and height for age and sex of the children. According to weight for age children were divided into 5 categories, that is, (1) obese ≥ +3 SD (standard deviation), (2) overweight ≥ +2 SD, (3) normal < +2 SD  →  −2 SD, (4) underweight ≤ −2 SD, and (5) severe underweight ≤ −3 SD. According to their height for age, children were divided into 5 categories, that is, (1) very tall ≥ +3 SD, (2) tall ≥ +2 SD, (3) normal < +2 SD  →  −2SD, (4) stunted ≤ −2 SD, and (5) severely stunted ≤ −3 SD. Study was conducted in August and September 2015. Informed consent was taken from the parents, and one of the parents was present during measurement of height and weight.

Name, age on the day of test, sex, hemoglobin levels, ferritin levels, weight in Kilogram, status of weight according to WHO *Z*-score chart for weight for age, height in centimeters (cm.), and status of height according to WHO *Z*-score chart for height for age were entered on a data form.

Shapiro–Wilk test was used to test the normality of continuous variables. Mean values ± standard deviation were used to show central tendency and spread of normally distributed data, and Median value (interquartile range [IQR]) was used to show central tendency and spread of nonnormal data. Chi-square test was used to test association. Spearman test was used to test correlation between two continuous variables. Value of Spearman coefficient (*r*_*s*_) from 0 to 0.19 was taken as very weak, from 0.20 to 0.39 as weak, from 0.40 to 0.59 as moderate, from 0.60 to 0.79 as strong, and from 0.80 to 1.0 as very strong correlation. Multiple linear regression analysis was applied to find out correlates of ferritin. For this purpose 3 models were made. In Model 1, ferritin level was taken as dependent variable, and status of height according to *Z*-score charts, age, and gender were taken as independent variables. In Model 2, ferritin was taken as dependent variable and status of weight according to *Z*-score charts, age, and gender were taken as independent variables. In Model 3, ferritin was taken as dependent variable and age, gender, status of weight according to *Z*- score, status of height according to *Z*-score charts, and anemia were taken as independent variables. Results were reported for these models as standardized beta coefficient (*β*) and level of significance (*p* value).

## 3. Results

One thousand and eighty students got admitted to prep class (first year of school) of five schools of Nazarat Taleem school system, in Rabwah. One in every four students was selected to be included in the study by computer randomization. One child had sore throat, so he was excluded from the study. Full blood count and ferritin levels of the remaining 295 children were checked. Out of these 295 children 164 (55.6%) were female and 131 (44.6%) were male. Among the continuous variables height had normal distribution. And age, weight, ferritin levels, and hemoglobin levels had nonnormal distribution. Median age was 67 (IQR: 9.0) months. Baseline characteristics and hemoglobin and ferritin levels of males, females, and all subjects included in the study are shown in Table 1. Out of 295, 240 (81.4%) had normal Hb and 55 (18.6%) had anemia. The difference between frequency of anemia among male and female subjects was nonsignificant (*p* = 0.26). See Table 2. Ferritin level was found to be normal in 49 (16.6%) children and it was found to be below normal level in 246 (83.4%) children. The difference between frequencies of low ferritin levels among male and female subjects was found to be nonsignificant (*p* = 0.47). See Table 2. Among those with anemia 81.8% had low ferritin levels, and among those with normal hemoglobin levels 83.4% had low ferritin levels. See Table 3. According to WHO *Z*-score weight for age chart 3 (1%), 238 (80.7%), 36 (12.2%), and 18 (6.1%) children were categorized as obese, having normal weight, underweight, and severely underweight, respectively. According to WHO *Z*-score height for age chart 2 (0.7%), 260 (88.1%), 26 (8.8%), and 7 (2.4%) children were categorized as tall, having normal height, stunted, and severely stunted, respectively.

Spearman test showed that there was very weak correlation between ferritin and hemoglobin levels (*r*_*s*_ = .163). Table 3 shows that median ferritin levels among the patients with normal hemoglobin and anemia were 5.1 ng/ml and 3.9 ng/ml, respectively. Mann–Whitney *U* test showed that the difference was nonsignificant. (*p* = 0.112)

Table 4 shows results of regression Model 1. None of the independent variables, that is, status of height, age, and gender had significant *p* value.  Table 5 shows results of regression Model 2. None of the independent variables, that is, status of height, age, and gender had significant *p* value.  Table 6 shows results of regression Model 3. None of the independent variables had significant *p* value. This shows that level of ferritin cannot be predicted by status of height and weight, presence of anemia, age, and gender.

## 4. Discussion

Iron deficiency anemia and iron deficiency without anemia are common nutritional problems in pediatric age group worldwide. A study by Killip et al. showed that, in Pakistan among children from 6 months to five years of age, 62.3% were anemic (hemoglobin levels <110 g/L), while 33.2% had iron deficiency anemia (defined as having both hemoglobin levels <110 g/L and ferritin levels <12 *μ*g/L). Ferritin deficiency (ferritin levels <12 *μ*g/L) was present in 47.1% cases, and 13.9% had ferritin deficiency without anemia [10, 11].

Our study focused on children who were starting first year of school. It showed that frequency of anemia was present in 18.6% cases, out of these 81.8% had low ferritin levels. Low ferritin levels were present in 88% of children, and 66.7% of the children included in this study had nonanemic iron deficiency (defined as ferritin < 10 ug/L). As compared with the study by Killip et al., our study showed low frequency of anemia and high frequency of iron deficiency. There can be different possible explanations for the difference in frequencies found in the two studies. Our study included only children starting the first year of school while the study by Killip et al. included the children from six months to five years of age. Our study included children from a single small urban community, and later study included children from a nationwide survey. It highlights the possibility that there can be communities and age groups with prevalence of anemia which is less than half the national average, but high frequency of low ferritin levels is present like silent hunger. Another study in Pakistan by Zeeshan et al., which included children presenting in a tertiary care hospital with anemia from 2 months to 2 years of age, showed that only 4% of the children had low ferritin levels, 60% had low folic acid, and 45% had low Vit B 12 levels [12]. It is worth noting that our study included patients with median age of 67 (IQR: 9.0) months; on the other hand the study by Zeeshan et al. included children from 2 months to 2 years of age. This difference can be a possible reason that in our study 81.8% anemic children had low ferritin levels, while in the study by Zeeshan et al. only 4% of the anemic children had low ferritin levels.

Our study showed that ferritin levels have got weak correlation with hemoglobin levels. Some other studies have analyzed correlation between hemoglobin and ferritin. Study by Khan et al. showed that among overweight and obese people, ferritin had negative correlation with Hb, iron, TIBC, and transferrin saturation (*p* < 0.001) [13]. Another study including pregnant women in third trimester showed that ferritin had weak correlation with hemoglobin (with Spearman coefficient of 0.21) [14]. Study by Kusumastuti et al. showed that, among children 6–59 months of age, ferritin had weak negative correlation with hemoglobin (Spearman constant = −0.220) [15].

The abovementioned studies show that correlation between ferritin and hemoglobin varies from being weak negative to weak positive. Despite the fact that iron deficiency is the commonest cause of anemia, according to our research no study has shown moderate or strong correlation between ferritin and hemoglobin levels. Keeping in view the fact that iron deficiency is the commonest cause of anemia and ferritin level is the measure of iron stores, more strong correlation is expected between ferritin and hemoglobin levels. Different possible causes can be considered responsible for this scenario. Study by Kate et al. showed that iron content of ferritin molecules varies under different conditions. This leads to the conclusion that merely ferritin level may not depict true status of iron stores [16]. Moreover release of iron from core of iron molecules is another step before iron is made available for use by different cells. Different factors can affect release of iron by ferritin molecules in vivo [17].

Some other studies have analyzed association of anemia and low ferritin levels with nutritional status. Study by Nodoshan et al. showed that children, from 6 to 60 months of age, who were having malnutrition according to Gomez classification, had significantly low hemoglobin, MCV, and MCH as compared with nutritionally normal children. Frequency of anemia was high among children with malnutrition. But there was no significant difference between ferritin levels and frequency of low ferritin levels among the two groups [18]. A meta-analysis, conducted among children from 6 months to 59 months of age, showed that anemia was associated with both underweight and stunting in majority of the studies [19]. Secondary analysis was performed on the National Nutrition Survey in Pakistan 2011-2012 conducted by Killip et al. which showed that stunting was associated with iron deficiency anemia among the children from 6 months to 5 years of age [10, 11]. Regression model of our study showed that ferritin level was not predictable by status of height and weight, gender, age, and presence of anemia.

It is important to study prevalence of iron and anemia at the start of school life because a number of studies have shown that children who had anemia or iron deficiency without anemia demonstrated psychomotor retardation, cognitive delays, lower cognitive scores later on, and lower academic performance, especially in mathematics [20–22].

Some studies have shown that early recognition and treatment of iron deficiency with or without anemia can reverse psychomotor delay and improves psychomotor development [23, 24]. On the other hand, other studies have shown that children who suffered chronic iron deficiency in infancy did not catch up to the group with good iron status in cognitive scores over time [20]. This highlights the importance of timely recognition and treatment of iron deficiency in early childhood. Otherwise this can lead to permanent consequences. Our study has shown very high frequency of low ferritin levels among children starting first year of their school life.

## 5. Conclusions

Low ferritin levels are frequent among children starting first year of school life. Low ferritin levels are equally frequent among children with or without anemia. There is weak correlation between ferritin and hemoglobin levels.

## Figures and Tables

**Table 1:** Baseline characteristics of males, females, and all subjects.

Variable	Males	Females	All subjects
Number (%)	139 (44.6%)	164 (55.6%)	295 (100%)
Median age in months (IQR)	67 (62–70)	67 (61–70)	67 (61–70)
Median weight in kilogram (IQR)	17.50 (16–19.50)	17.0 (15–19)	17.0 (15.5–19.0)
Mean height in centimeter (±SD)	109.02 (±6.01)	108.01 (±5.29)	108.4 (±5.63)
Median Hb gm/dl (IQR)	11.90 (11.50–12.50)	12.10 (11.50–12.78)	12.0 (11.5–12.7)
Median ferritin ng/ml (IQR)	4.40 (2.8–7.3)	5.10 (3.03–7.30)	4.90 (4.9–12.4)

**Table 2:** Frequency of anemia and low ferritin in males, females, and all subjects.

	Males	Females	Total	*p* value
Normal hemoglobin level	104 (79.4%)	136 (82.9%)	240 (81.3%)	0.26
Anemia	27 (20.6%)	28 (17.1%)	55 (18.7%)	
Normal ferritin levels	21 (16%)	28 (17%)	49 (16.6%)	0.47
Low ferritin levels	110 (84%)	136 (83%)	246 (83.4%)	

**Table 3:** Status of ferritin among subjects with anemia and normal hemoglobin.

Status of hemoglobin	Normal ferritin	Low ferritin
Normal hemoglobin	39 (16.2%)	201 (83.8%)
Anemic	10 (18.2%)	45 (81.8%)

*p* = 0.72.

**Table 4:** Linear regression Model 1. Ferritin as dependent variable and status of height, age, and gender as independent variables.

Factor	Standardized coefficient beta	*p* value
Status of height according to *Z*-score chart	−.028	.634
Age	.044	.454
Gender	.067	.251

**Table 5:** Linear regression Model 2. Ferritin as dependent variable and status of weight, age, and gender as independent variables.

Factor	Standardized coefficient beta	*p* value
Status of weight according to *Z*-score charts	.051	.382
Age	.043	.466
Gender	.07	.223

**Table 6:** Linear regression Model 3. Ferritin as dependent variable and anemia, status of height, status of weight, age, and gender as independent variable.

Factor	Standardized coefficient beta	*p* value
Anemia	−.084	.156
Status of height according to *Z*-score charts	−.059	.352
Status of weight according to *Z*-score charts	.083	.193
Age	.052	.377
Gender	.070	.234

## Data Availability

Data file is available.

## Additional Points

*Key Points*. (i) Iron deficiency is very common among children starting school. Iron deficiency without anemia is more common as compared with iron deficiency with anemia. (ii) Ferritin level cannot be predicted by independent variables like status of height, weight, age, and anemia.
